# MicroRNA-494 promotes apoptosis and extracellular matrix degradation in degenerative human nucleus pulposus cells

**DOI:** 10.18632/oncotarget.15838

**Published:** 2017-03-02

**Authors:** Liang Kang, Cao Yang, Yu Song, Kangcheng Zhao, Wei Liu, Wenbin Hua, Kun Wang, Ji Tu, Shuai Li, Huipeng Yin, Yukun Zhang

**Affiliations:** ^1^ Department of Orthopaedics, Union Hospital, Tongji Medical College, Huazhong University of Science and Technology, Wuhan 430022, China; ^2^ Department of Orthopaedics, First Hospital of Wuhan, Wuhan 430022, China

**Keywords:** intervertebral disc degeneration, nucleus pulposus, miR-494, SOX9, methylation

## Abstract

**Purpose:**

This study investigated the expression and function of the microRNA-494 in intervertebral disc degeneration (IDD).

**Results:**

MicroRNA-494 expression was upregulated during IDD progression; its overexpression increased the expression of ECM catabolic factors such as matrix metalloproteinase and A disintegrin and metalloproteinase with thrombospondin motif in NP cells while decreasing that of anabolic genes such as type II collagen and aggrecan; it also induced the apoptosis of NP cells, as determined by flow cytometry. These effects were reversed by microRNA-494 inhibitor treatment. SOX9 was identified as a target of negative regulation by microRNA-494. Promoter hypomethylation and NF-κB activation were associated with microRNA-494 upregulation in IDD.

**Materials and Methods:**

MicroRNA-494 expression in degenerative nucleus pulposus (NP) tissue was assessed by quantitative real-time PCR. The effect of microRNA-494 on extracellular matrix (ECM) metabolism and NP cell apoptosis was evaluated by transfection of microRNA-494 mimic or inhibitor. The regulation of SRY-related high mobility group box (SOX)9 expression by microRNA-494 was assessed with the luciferase reporter assay, and the methylation status of the microRNA-494 promoter was evaluated by methylation-specific PCR and bisulfite sequencing PCR. The role of activated nuclear factor (NF)-κB in the regulation of microRNA-494 expression was evaluated using specific inhibitors.

**Conclusions:**

MicroRNA-494 promotes ECM degradation and apoptosis of degenerative human NP cells by directly targeting SOX9.

## INTRODUCTION

Chronic low back pain (CLBP) is a common musculoskeletal disorder affecting up to 80% of individuals at some point during their lifetime, placing an economic burden on society [1, 2]. Intervertebral disc degeneration (IDD) is the major cause of CLBP [3]. IDD has a complex pathogenesis that is influenced by multiple risk factors, including genetics, lifestyle, and aging [4, 5]. Intervertebral discs are composed of the nucleus pulposus (NP), annulus fibrosus (AF), and cartilage end plates. NP cells maintain the homeostasis of the extracellular matrix (ECM), which includes type II collagen and aggrecan [6]. Excessive apoptosis of NP cells is an initiating event in IDD [7]. In addition, decreases in type II collagen and aggrecan levels—which arise due to an imbalance between ECM anabolism and catabolism—and consequent ECM degradation are features of IDD [8]. However, the mechanisms underlying these processes remain poorly understood.

Micro (mi)RNAs are short (20–25 nucleotides) non-coding RNA molecules that silence gene expression by binding to the 3′-untranslated region (3′-UTR) of target mRNAs, leading to translational repression or mRNA degradation [9]. Approximately one-third of all mammalian genes are controlled by miRNAs in various cellular processes including proliferation, apoptosis, and differentiation [10, 11]. Aberrant miRNA expression is linked to the development and progression of IDD [12, 13]. For example, the overexpression of miR-100 promotes lumbar disc degeneration by activating matrix metalloproteinase (MMP)13 [14]. MiR-193a-3p regulates MMP14 expression and type II collagen degradation in human NP cells [12]. MiR-155 is downregulated in degenerative human NP cells, which increases apoptosis by targeting Fas-associated protein with death domain and caspase-3 [15]. On the other hand, miR-21 stimulates the proliferation of degenerative human NP cells by targeting Programmed cell death 4 [16]. Thus, miRNAs play important roles in the metabolism of degenerative NP cells, including apoptosis and ECM degradation; clarifying the underlying mechanisms can provide insight into the pathogenesis of IDD as well as a basis for developing effective therapeutic strategies for IDD treatment.

MiR-494 was previously shown to be upregulated in degenerative human NP cells [17] and has been implicated in various pathological conditions through regulation of cell proliferation, apoptosis, and ECM degradation [18, 19]. However, the role of miR-494 in the pathogenesis of IDD is not well understood. Using miRNA target prediction algorithms, we identified a putative miR-494 binding site in the 3′-UTR of human SRY-related high mobility group box (SOX)9, a critical regulator of chondrogenesis [20]. Previous studies have shown that SOX9 expression is downregulated in degenerated discs, which is associated with IDD [21, 22]. In addition, type II collagen expression was increased in NP cells overexpressing SOX9 [23]. SOX9 was also found to protect against cell apoptosis induced by interleukin (IL)-1β [24].

Based on the above findings, we hypothesized that miR-494 may be involved in IDD development via direct targeting of SOX9. We found that miR-494 overexpression promotes apoptosis of degenerative human NP cells and ECM degradation via negative regulation of SOX9. We also demonstrate that aberrant miR-494 expression is partly controlled by hypomethylation of its promoter region and activation of nuclear factor (NF)-κB in IDD. These findings provide mechanistic insight into the role of miR-494 in IDD pathogenesis.

## RESULTS

### MiR-494 expression level is correlated with IDD grade in degenerative human NP tissue

**Figure 1:**
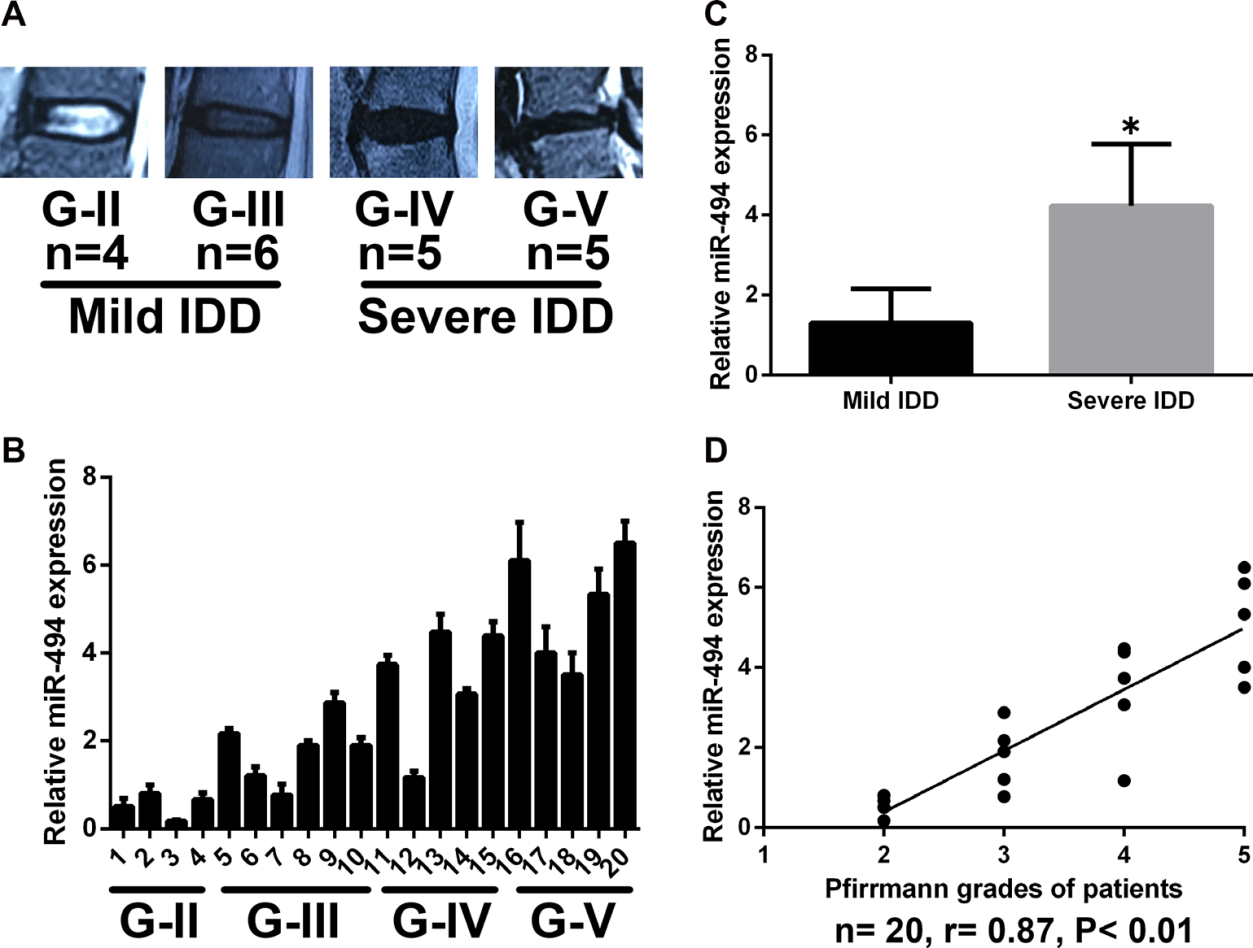
MiR-494 expression in human NP tissue (**A**) Disc tissue from patients were collected and classified according to the modified Pfirrmann grading system; grades II and III (*n* = 10) were designated as mild IDD, while grades IV and V (*n* = 10) were designated as severe IDD. (**B**) MiR-494 expression in human NP tissue (*n* = 20), as determined by qRT-PCR. (**C**) MiR-494 level was lower in mild as compared to severe IDD (*n* = 10 each). (**D**) Correlation between miR-494 expression and Pfirrmann grade (*n* = 20). U6 was used as an internal control. Data represent mean ± SD. *P < 0.05 vs. mild IDD group.

The degree of IDD was graded based on results of the magnetic resonance imaging (MRI) according to the modified Pfirrmann grading system (Figure 1A). Samples were divided into two groups: mild IDD (grades II and III) and severe IDD (grades IV and V) (*n* = 10 each). The expression of miR-494 in degenerative human NP tissue was examined by qRT-PCR (Figure 1B). We found that miR-494 expression was higher in NP tissue from severe as compared to mild IDD (Figure 1C); furthermore, miR-494 level was positively correlated with disc degeneration grade (Figure 1D).

### MiR-494 promotes ECM degradation

**Figure 2:**
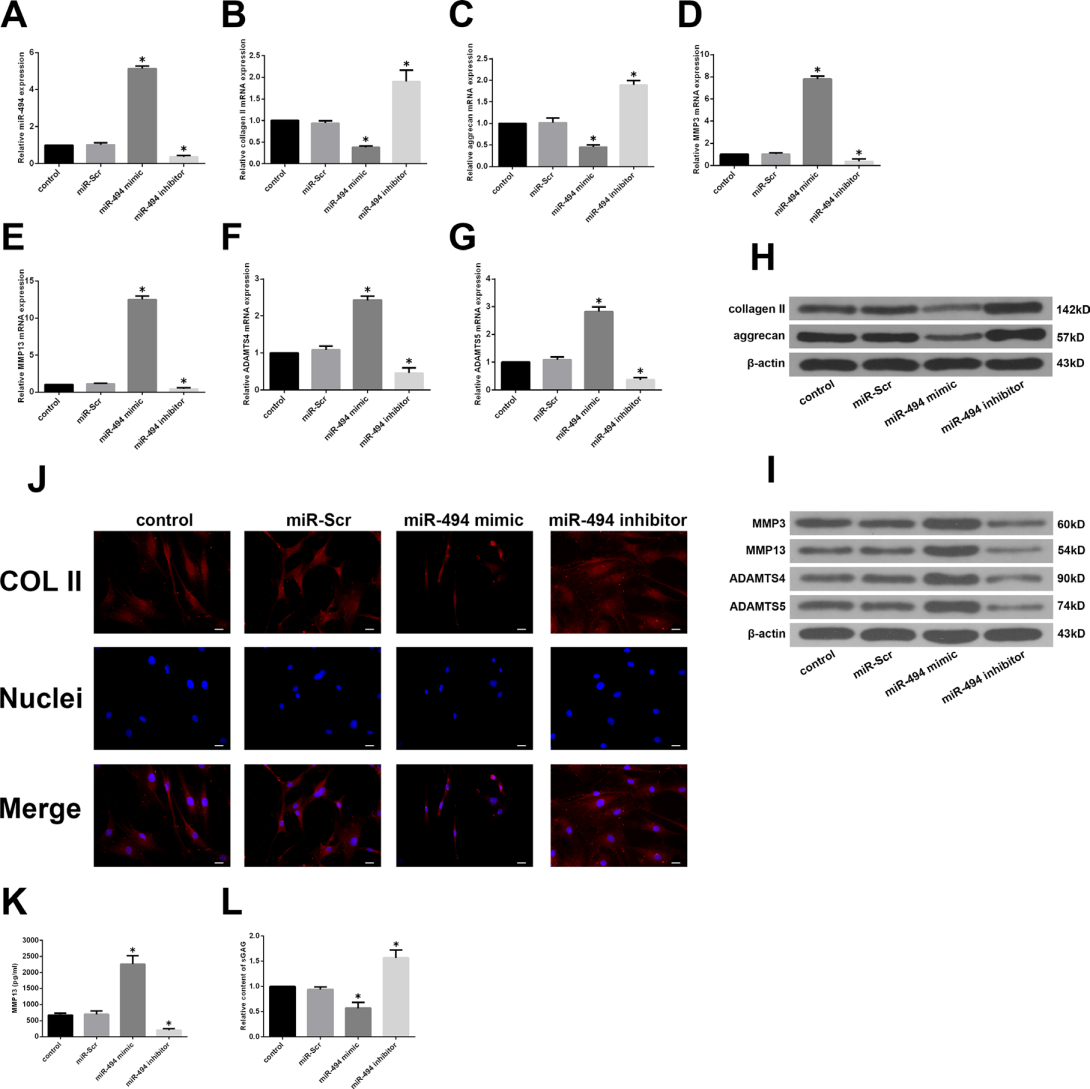
Effect of miR-494 on ECM degradation of degenerative human NP cells NP cells were transfected with miR-Scr (100 nM), miR-494 mimic (100 nM), or miR-494 inhibitor (100 nM) for 48 h, with untransfected cells serving as a control. (**A**) MiR-494 level was measured by qRT-PCR. U6 was used as an internal control. (**B**–**I**) Type II collagen, aggrecan, MMP3, MMP13, ADAMTS4, and ADAMTS5 mRNA (B–G) and protein (H, I) expression levels, as determined by qRT-PCR and western blotting, respectively; β-actin was used as a control. (**J**) Immunofluorescence staining of type II collagen in transfected NP cells. Scale bars: 20 μm. (**K**) The protein expression of MMP13 in extracellular matrix of NP cells was measured by ELISA. (**L**) sGAG concentrations in NP cells were determined by DMMB assay. Data represent mean ± SD. *P < 0.05 vs. control group.

To investigate the effect of miR-494 on degenerative human NP cells, miR-494 mimic, miR-494 inhibitor, or miR-Scr were transfected into the cells and the expression of ECM anabolic genes was assessed by qRT-PCR and western blotting. The efficient transfection of miR-494 was confirmed by qRT-PCR (Figure 2A). Transfection of miR-494 mimic decreased whereas miR-494 inhibitor increased the expression of type II collagen and aggrecan relative to the control (Figure 2B, 2C and 2H); this effect was confirmed by immunofluorescence staining (Figure 2J). ECM catabolic proteinases such as MMPs and A disintegrin and metalloproteinase with thrombospondin motifs (ADAMTSs) are highly expressed in degenerative intervertebral disc tissue and cells, and have been linked to ECM degradation and IDD progression [25]; we therefore evaluated the expression of MMP3, MMP13, ADAMTS4, and ADAMTS5 in NP cells. MiR-494 overexpression increased the mRNA and protein levels of all four factors, whereas miR-494 inhibition had the opposite effect (Figure 2D–2G and 2I). Moreover, the results of enzyme-linked immunosorbent assay (ELISA) showed that the variation tendency of MMP13 protein expression in extracellular matrix of NP cells transfected with mimic or inhibitor of miR-494 (Figure 2K) was in accordance with that of intracellular MMP13 protein expression (Figure 2I). Additionally, the concentrations of sulfated glycosaminoglycan (sGAG), a main form of aggrecan secreted by NP cells in the intervertebral disc, declined in NP cells transfected with 494 mimic, while miR-494 inhibitor upregulated the concentrations of sGAG using 1,9-dimethylmethylene blue (DMMB) assay (Figure 2L), similar to the variation tendency of aggrecan using western blotting (Figure 2H). These results indicate that miR-494 induces ECM degradation in degenerative human NP cells.

### MiR-494 enhances apoptosis of degenerative human NP cells

**Figure 3:**
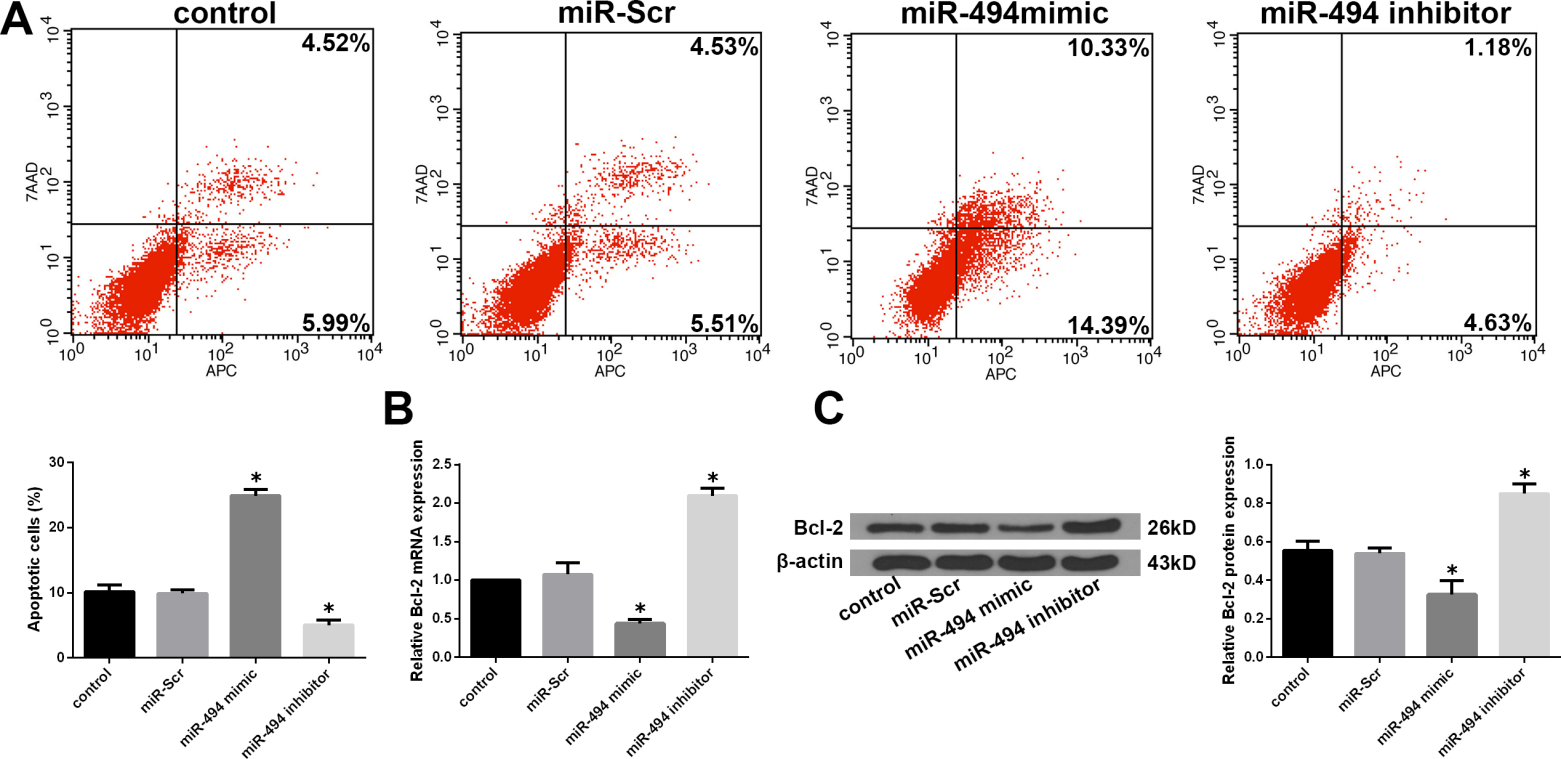
Effect of miR-494 on apoptosis of degenerative human NP cells NP cells were transfected with miR-Scr, miR-494 mimic, or miR-494 inhibitor for 48 h, with untransfected cells serving as a control. (**A**) Rate of apoptosis in degenerative human NP cells, as determined by flow cytometry. (**B**) *Bcl-2* mRNA level, as determined by qRT-PCR. (**C**) Bcl-2 protein level, as determined by western blotting; β-actin was used as a loading control. Data represent mean ± SD. *P < 0.05 vs. control group.

Given that NP cell apoptosis plays a major role in the development of IDD [7], we investigated whether miR-494 modulates degenerative human NP cell apoptosis. Flow cytometry analysis revealed that transfection of miR-494 mimic increased whereas miR-494 inhibitor decreased the rate of apoptosis as compared to the control (Figure 3A). Meanwhile, B cell lymphoma (Bcl)-2 expression was down- and upregulated in cells transfected with miR-494 mimic and inhibitor, respectively, as determined by qRT-PCR and western blotting (Figure 3B and 3C). These results demonstrate that miR-494 overexpression induces apoptosis of degenerative human NP cells.

### SOX9 is a direct target of miR-494

**Figure 4:**
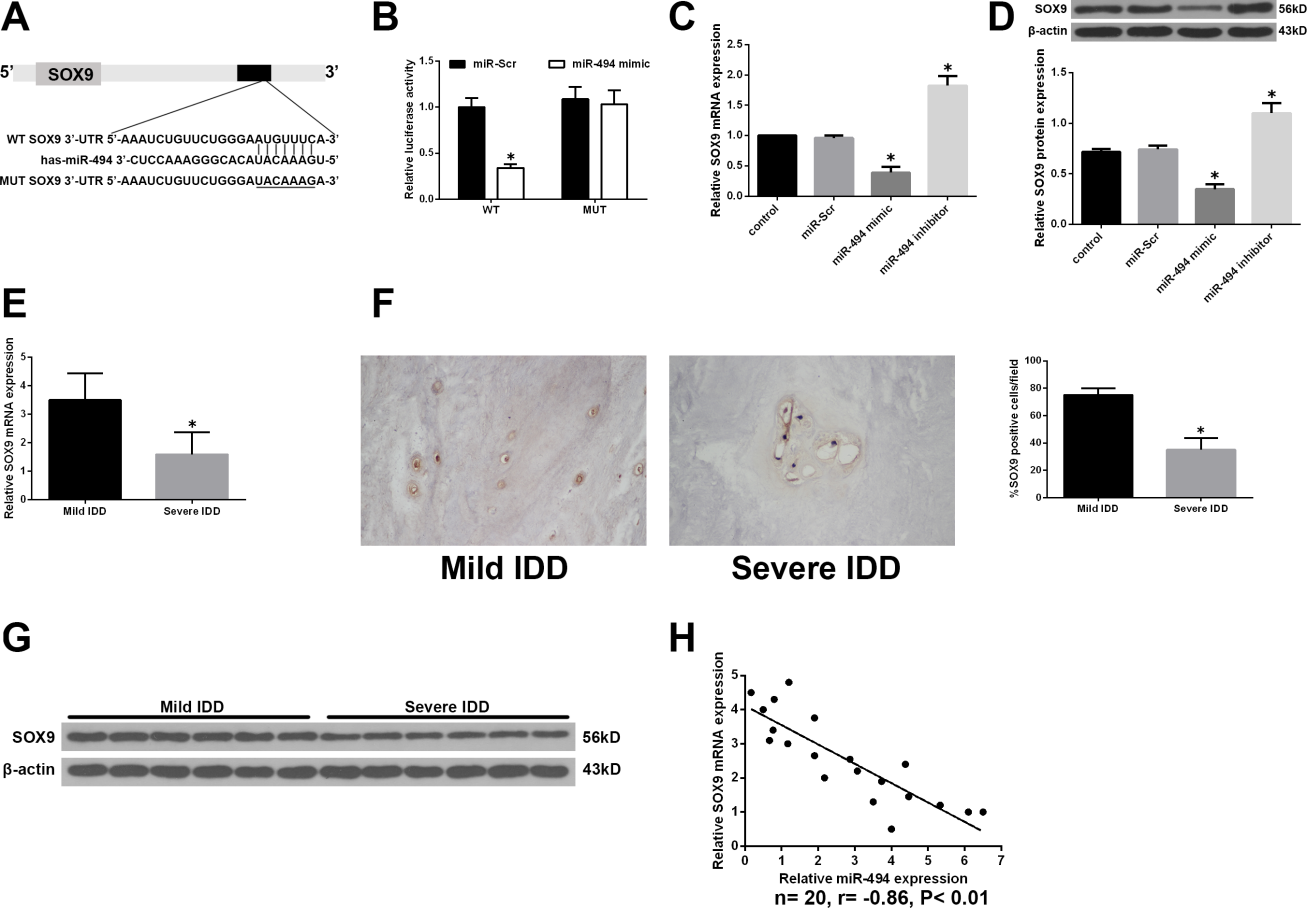
SOX9 is a direct target of miR-494 (**A**) Putative miR-494 target site in the 3′-UTR of human SOX9 transcript predicted by bioinformatics analysis. (**B**) Luciferase activity in HEK 293 cells co-transfected with miR-494 mimic or miR-Scr and WT or MUT SOX9 3′-UTR constructs. *P < 0.05 vs. miR-Scr group. (**C**, **D**) NP cells were transfected with miR-494 mimic, miR-494 inhibitor, or miR-Scr for 48 h, with untreated cells serving as a control. (C) *SOX9* mRNA level, as determined by qRT-PCR. (D) SOX9 protein level, as determined by western blotting. β-actin was used as a loading control. *P < 0.05 vs. control group. (**E**) SOX9 transcript level in human NP tissue samples from mild IDD (*n* = 10) and severe IDD (*n* = 10) groups, as determined by qRT-PCR. (**F**) Representative images of SOX9 protein expression in NP tissue (*n* = 20) was detected by immunohistochemistry. Magnification: 400×. (**G**) SOX9 protein level in NP tissue, as determined by western blotting. (**H**) Correlation between *SOX9* mRNA and miR-494 levels in human NP tissue (*n* = 20); β-actin was used as loading control. Data represent mean ± SD. *P < 0.05 vs. mild IDD group.

To clarify the mechanism by which miR-494 induces ECM degradation and apoptosis of degenerative human NP cells, we used a bioinformatics approach to search for miR-494 target genes and found that the 3′-UTR of SOX9 contains sequences that are complementary to the miR-494 seed sequence (Figure 4A). To determine whether SOX9 is direct target of miR-494, we generated luciferase reporter vectors containing the wild-type (WT) or mutant (MUT) 3′ UTR of SOX9. Luciferase activity was markedly suppressed relative to the miR-Scr group by co-transfection of miR-494 mimic and the WT 3′-UTR of SOX9, while co-transfection of the SOX9 MUT 3′-UTR abrogated this effect (Figure 4B). We also found that miR-494 overexpression reduced SOX9 mRNA and protein levels in degenerative NP cells (Figure 4C and 4D). The converse was observed by inhibiting miR-494. Moreover, SOX9 mRNA level was lower in NP from severe as compared to mild IDD tissue (Figure 4E); the same trend was observed for SOX9 protein, as determined by western blotting and immunohistochemistry (Figure 4F and 4G). The expression levels of SOX9 and miR-494 were negatively correlated (Figure 4H). These results provide evidence that SOX9 is a direct target of miR-494.

### MiR-494 exerts its functions by targeting SOX9 in degenerative human NP cells

**Figure 5:**
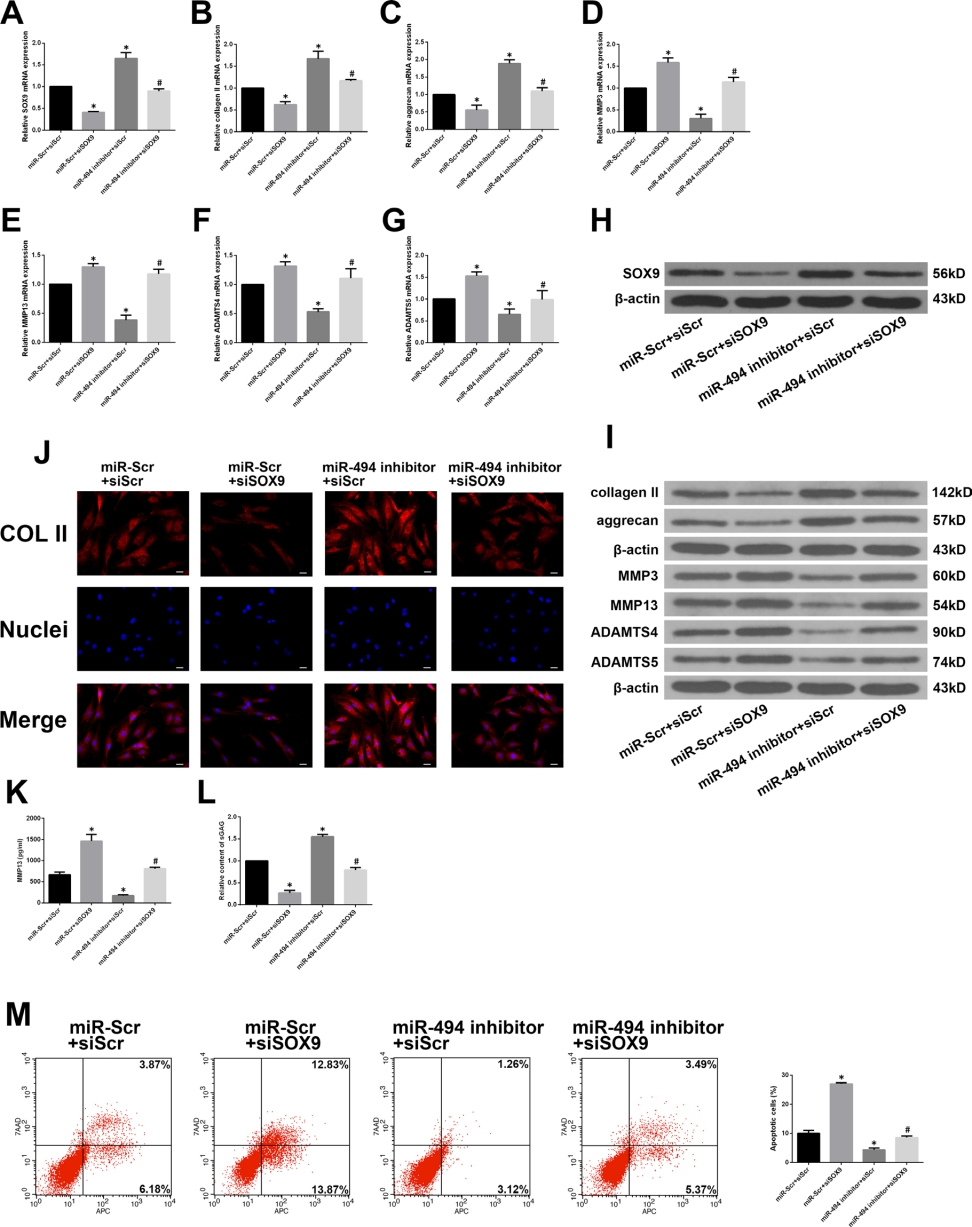
MiR-494 induces ECM degradation and degenerative human NP cell apoptosis by targeting SOX9 (**A**) SOX9 mRNA level in NP cells co-transfected with miR-494 inhibitor (100 nM) or miR-Scr (100 nM) and siSOX9 (100 nM) or siScr (100 nM), as determined by qRT-PCR. (**B**–**G**) Type II collagen, aggrecan, MMP3, MMP13, ADAMTS4, and ADAMTS5 mRNA expression in transfected NP cells, as determined by qRT-PCR. (**H**, **I**) SOX9 (H), type II collagen, aggrecan, MMP3, MMP13, ADAMTS4, and ADAMTS5 (I) protein expression in transfected NP cells, as determined by western blotting. (**J**) Immunofluorescence analysis of type II collagen expression in transfected NP cells. Scale bars: 20 μm. (**K**) The protein expression of MMP13 in extracellular matrix of NP cells was measured by ELISA. (**L**) sGAG concentrations in NP cells were determined by DMMB assay. (**M**) Rate of apoptosis of transfected NP cells, as determined by flow cytometry. β-actin was used as an internal control. Data represent mean ± SD. *P < 0.05 vs. miR-Scr + siScr group; ^#^*P* < 0.05 vs. miR-494 inhibitor + siScr group.

To determine whether ECM degradation and degenerative human NP cell apoptosis induced by miR-494 result from its direct targeting of SOX9, we co-transfected the cells with miR-494 inhibitor or miR-Scr along with short interfering (si)RNA against SOX9 (siSOX9) or siScr. SOX9 expression was markedly suppressed by siSOX9; the upregulation of SOX9 after transfection with miR-494 inhibitor was abrogated by co-transfection of siSOX9 (Figure 5A and 5H). Furthermore, inhibiting miR-494 increased type II collagen and aggrecan and decreased MMP-3, MMP13, ADAMTS4, and ADAMTS5 mRNA and protein levels, the effect was reversed by siSOX9 (Figure 5B–5G and 5I). We evaluated the protein level of type II collagen in transfected cells by immunofluorescence. Similar differences in type II collagen level were observed between NP cells transfected with miR-494 inhibitor alone and those co-transfected with siSOX9 by immunofluorescence (Figure 5J) and western blotting (Figure 5I). The results of ELISA showed that co-transfection with miR-494 inhibitor and siSOX9 decreased MMP13 protein expression compared with transfection with miR-494 inhibitor alone (Figure 5K). An increase in sGAG content was observed after transfection with the miR-494 inhibitor alone, however, the increase was blocked by co-transfection with miR-494 inhibitor and siSOX9 (Figure 5L). Moreover, flow cytometry analysis showed that siSOX9 transfection increased the incidence of degenerative NP cell apoptosis; the rate was even higher in cells that were co-transfected with siSOX9 and miR-494 inhibitor as compared to the latter alone (Figure 5M). These results suggest that SOX9 plays an essential role in miR-494-induced ECM degradation and apoptosis in degenerative human NP cells.

### MiR-494 expression is regulated by methylation of CpG islands in the promoter region

**Figure 6:**
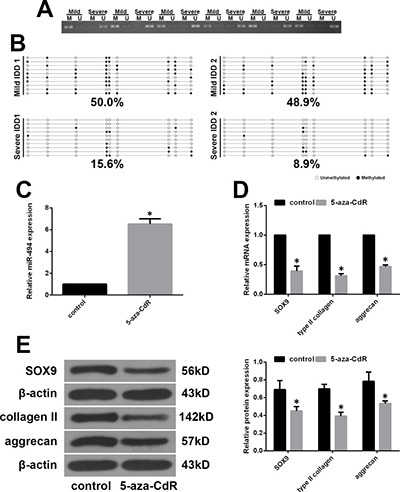
MiR-494 expression is regulated by promoter methylation status (**A**) Representative results of MSP analysis of miR-494 in human NP tissue from mild and severe IDD groups. M, methylated primers; U, unmethylated primers. (**B**) Representative results of BSP of miR-494 methylation in human NP tissue from mild and severe IDD groups. Black and white circles represent methylated and unmethylated CpG sites, respectively. (**C**–**E**) Mild IDD NP cells were treated with 5 μM 5-aza-CdR for 3 days, and untreatedcells were used as a control. (C) MiR-494 expression in mild IDD NP cells, as determined by qRT-PCR. U6 was used as an internal control. (D, E) Transcript (D) and protein (E) levels of SOX9, type II collagen, and aggrecan in mild IDD NP cells, as determined by qRT-PCR and western blotting, respectively. β-actin was used as a control. Data represent mean ± SD. *P < 0.05 vs. control group.

Epigenetic modifications such as DNA methylation in the promoter are thought to regulate miRNA expression [26, 27]. To clarify the mechanism underlying the aberrant expression of miR-494 in IDD, we compared the methylation status of the miR-494 promoter in mild and severe IDD NP tissue samples by methylation-specific PCR (MSP). Promoter methylation was higher in mild than in severe IDD, corresponding to the lower miR-494 expression in the former relative to the latter (Figure 6A). The methylation status of CpG sites in the miR-494 promoter was measured in 20 NP tissue samples by bisulfite sequencing PCR (BSP). Consistent with the results obtained by MSP, DNA methylation levels were higher in mild as compared to severe IDD NP tissue (Figure 6B). To confirm these findings, we treated mild IDD NP cells with the demethylating agent 5-aza-2′-deoxycytidine (5-aza-CdR), and found that miR-494 expression was increased (Figure 6C). We speculated that miR-494 targets would be downregulated in cells treated with 5-aza-CdR. To test this hypothesis, we evaluated SOX9, type II collagen, and aggrecan levels in mild IDD NP cells treated with 5-aza-CdR by qRT-PCR and western blotting. We found that the expression of all three factors was reduced relative to their respective levels in untreated cells (Figure 6D and 6E). These findings indicate that aberrant expression of miR-494 in IDD is due to hypomethylation of its promoter region.

### Activation of NF-κB signalling induces miR-494 expression

**Figure 7:**
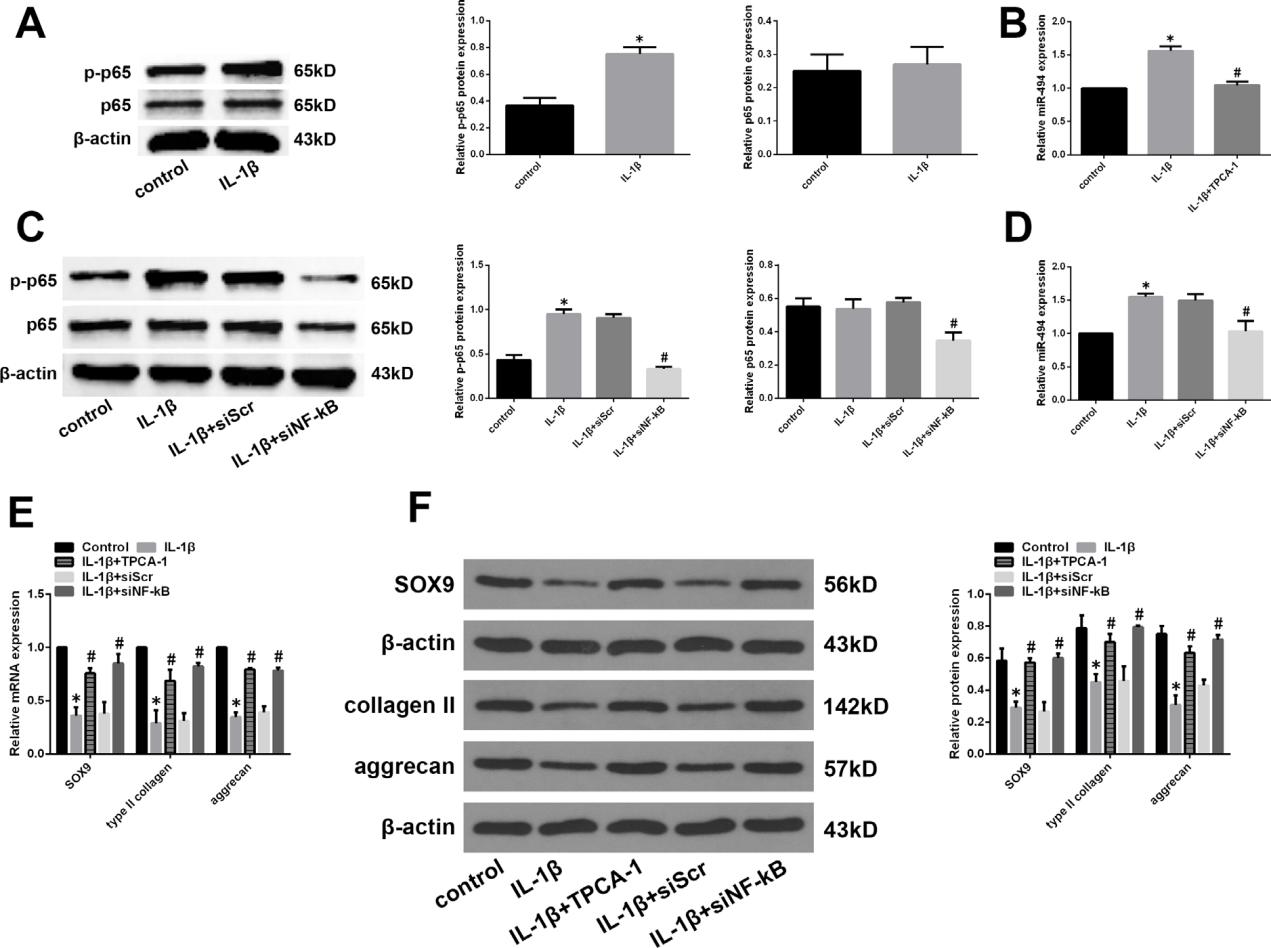
Role of NF-κB signalling in the regulation of miR-494 expression (**A**) Phosphorylated (p-) and total protein levels of NF-κB p65 in mild IDD NP cells treated with IL-1β (10 ng/ml) for 24 h, as determined by western blotting. (**B**) Mild IDD NP cells were pretreated for 2 h with the IKK inhibitor TPCA-1 (30 μM), then stimulated with IL-1β for 24 h. MiR-494 expression was evaluated by qRT-PCR; untreated NP cells served as a control. β-actin and U6 were used as internal controls for phosphorylated NF-κB p65 and miR-494 levels, respectively. *P < 0.05 vs. control group; ^#^*P* < 0.05 vs. IL-1β stimulation only group. (**C**, **D**) Mild IDD NP cells were transfected with siNF-κB (100 nM) or siScr (100 nM), then stimulated with IL-1β (10 ng/ml) for 24 h; untreated NP cells served as a control. Phosphorylated (p-) and total protein levels of NF-κB p65 (C) and miR-494 (D) expression was determined by western blotting and qRT-PCR, respectively, with β-actin and U6 used as the respective control. *P < 0.05 vs. control group; ^#^*P* < 0.05 vs. IL-1β stimulation only group. (**E**, **F**) Mild IDD NP cells were treated with TPCA-1 or siNF-κB, then stimulated with IL-1β; untreated NP cells served as a control. SOX9, type II collagen, and aggrecan mRNA (E) and protein (F) expression was evaluated by qRT-PCR and western blotting, respectively. β-actin was used as a control. Data represent mean ± SD. *P < 0.05 vs. control group; ^#^*P* < 0.05 vs. IL-1β stimulation only group.

Previous studies have reported that the NF-κB signalling pathway is aberrantly activated in IDD [28–30]; NF-κB signalling has been shown to regulate the expression of certain miRNAs [31, 32]. We therefore investigated whether NF-κB signalling regulates miR-494 expression in IDD. The NF-κB pathway was activated by application of IL-1β in degenerative NP cells. A western blot analysis revealed that IL-1β treatment increased the level of phosphorylated NF-κB p65, indicating activation of NF-κB signalling (Figure 7A). We also found that IL-1β treatment increased miR-494 expression; however, this effect was blocked by treatment with the IκB kinase (IKK) inhibitor 2-[(aminocarbonyl)amino]-5-(4-fluorophenyl)-3-thiophenecarboxamide (TPCA-1) (Figure 7B). IKK is an essential kinase in the NF-κB signalling pathway. We confirmed this effect in degenerative NP cells treated with short interfering (si)RNA against NF-κB (siNF-κB) or siScr and IL-1β. SiNF-κB suppressed the upregulation of phospho-p65 and miR-494 induced by IL-1β (Figure 7C and 7D). In addition, we assessed the changes in the expression of miR-494 targets. The downregulation of SOX9, type II collagen, and aggrecan mRNA and protein induced by IL-1β was reversed by TPCA-1 or siNF-κB treatment (Figure 7E and 7F). These results indicate that miR-494 expression is regulated by NF-κB signalling in IDD.

## DISCUSSION

MiRNAs have essential roles in the pathogenesis of several diseases and are therefore promising therapeutic targets [12, 19]. The role of miRNAs in degenerative human disc disease has also been investigated [12, 15]. Many miRNAs are differentially expressed in degenerative NP tissue [13, 14]; one study found that 25 miRNAs were upregulated and 26 were downregulated in IDD as compared to spinal cord injury specimens [33]. In this study, we found that miR-494 expression was higher in NP tissue from severe as compared to mild IDD and was positively correlated with disc degeneration grade.

IDD is the pathological basis of spinal degenerative diseases [3]. Although the molecular mechanisms underlying IDD are not fully understood, it is well established that apoptosis, ECM degradation, cell proliferation, and the inflammatory response contribute to IDD development [7, 8]. Some miRNAs that regulate these processes have been linked to IDD [16, 34, 35]. For example, miR-27b causes the loss of type II collagen by directly targeting MMP13, leading to IDD development [36]. MiR-146a suppresses IL-1β-induced MMP-13, ADAMTS4, and ADAMTS-5 expression in NP cells [34], and miR-98 downregulation contributes to the loss of type II collagen in IDD [35]. In this study, we found that miR-494 overexpression increased the levels of MMP3, MMP13, ADAMTS4, and ADAMTS5 and decreased that of type II collagen, aggrecan and sGAG; these effects were reversed by inhibiting miR-494. Additionally, we demonstrated that miR-494 overexpression induces degenerative NP cell apoptosis, which is similar to the pro-apoptotic effect of other miRNAs in NP cells. For instance, miR-27a is highly expressed in degenerative human NP cells and induces their apoptosis by blocking phosphoinositide 3-kinase/AKT signaling [37], while downregulation of miR-210 may promote Fas-mediated NP cell apoptosis in human IDD [38]. Moreover, our findings are also consistent with the results from the work by Wang et al. [39], which reported that miR-494 inhibition protects NP cells from TNF-α-induced apoptosis by targeting JunD.

It is well known that miRNAs exert their functions by directly binding to target transcripts to silence gene expression. We therefore used a bioinformatics approach to predict miR-494 targets and identified a putative miR-494 binding site in the 3′-UTR of SOX9, and found that miR-494 directly binds to this site. Furthermore, miR-494 overexpression and inhibition suppressed and stimulated, respectively, SOX9 mRNA and protein expression, indicating that SOX9 is a direct target of miR-494 in degenerative NP cells.

SOX9 is an essential transcription factor for chondrogenesis and type II collagen synthesis, and is considered as a master regulator of the chondrocyte phenotype [20]. SOX9 overexpression in degenerative disc cells increased type II collagen expression *in vitro*, and in a rabbit model, discs injected with adenovirus expressing SOX9 maintained a chondrocytic phenotype for 5 weeks [40]. SOX9 is associated with regulation of aggrecan expression in NP cells [41], meanwhile, it has been demonstrated that SOX9 overexpression suppressed MMP3, MMP13, ADAMTS4 and ADAMTS5 expression in chondrocytes [24, 42, 43]. Based on our findings that miR-494 was upregulated in NP cells from severe IDD cases and that SOX9 is a direct target of miR-494, we speculated that downregulation of SOX9 in IDD could be due to enhanced miR-494 expression. Indeed, we observed a downregulation of SOX9 expression in severe as compared to mild IDD and an inverse correlation between miR-494 and SOX9 levels in degenerative NP cells. We also found that SOX9 knockdown reversed the effects of miR-494 inhibition on the expression of ECM markers such as type II collagen, aggrecan, sGAG, MMP3, MMP13, ADAMTS4, and ADAMTS5 and the rate of apoptosis in degenerative NP cells. Thus, negative regulation of SOX9 by miR-494 contributes to ECM degradation and degenerative NP cell apoptosis in IDD.

DNA methylation is closely associated with the regulation of gene expression. Several studies have shown that abnormal methylation of the promoter region can lead to aberrant miRNA expression in human diseases [26, 27]. We therefore investigated whether upregulation of miR-494 in degenerative NP tissue is the result of promoter hypomethylation. Methylation levels were higher in mild than in severe IDD NP tissue, consistent with the higher expression level of miR-494 in the latter. Moreover, treatment with a demethylating agent increased miR-494 and decreased SOX9, type II collagen, and aggrecan expression in mild IDD NP cells, indicating that miR-494 is upregulated in IDD due to promoter hypomethylation.

NF-κB is a family of transcription factors that are activated in response to inflammation, cellular damage, and stress [44]. NF-κB signalling has been implicated in IDD pathogenesis [29]. It has been reported that activation of the NF-κB pathway occurs in intervertebral discs and especially in NP tissue, and that this is correlated with oxidative stress, and increases with age and tissue degeneration [28]. Activation of the NF-κB pathway in NP cells induces the expression of MMPs and ADAMTSs, which contribute to ECM catabolism in IDD [30]. NF-κB is also involved in the regulation of miRNA expression [31, 32]; in fact, three NF-κB binding sites have been identified in the 5′ flanking sequence of miR-494 [45]. It is therefore possible that the increased miR-494 expression in IDD is associated with activation of NF-κB signalling, which was found to be required for upregulation of miR-494 expression in the present study. In addition, miR-494 level was reduced whereas those of SOX9, type II collagen, and aggrecan were enhanced in degenerative NP cells treated with two different NF-κB inhibitors. These results indicate that activation of NF-κB signalling increases miR-494 expression in IDD.

There were some limitations to this study. We used mild degenerative NP tissue samples (grades II and III) instead of non-degenerative tissue as a control. There is no gold standard for diagnosing IDD. Several researchers have found that IDD occurs naturally with age [46]; it is therefore difficult to determine whether intervertebral disc samples are non-degenerated. However, we did observe some differences between the mild and severe IDD groups.

In conclusion, we demonstrated that promoter hypomethylation and activation of NF-κB signalling induced upregulation of miR-494 expression in IDD. MiR-494 in turn promoted ECM degradation and apoptosis of degenerative human NP cells by direct targeting SOX9. Therefore, strategies to downregulate the expression or to prevent the upregulation of miR-494 may have the potential to become a possible therapeutic and/or preventive approach for human IDD.

## MATERIALS AND METHODS

### Patient tissue samples

The study protocol was approved by the Ethics Committee of Tongji Medical College, Huazhong University of Science and Technology. Written, informed consent was obtained from all participants. Degenerative NP samples were obtained from 20 patients (*n* = 9 females and 11 males; mean age: 46.5 years, range: 30–58 years) with degenerative disc disease undergoing disc resection surgery or spinal fusion to relieve CLBP. The degree of IDD was assessed according to the modified Pfirrmann grading system [47] by pre-operative magnetic resonance imaging (MRI) scans. Grade II (*n* = 4) and III (*n* = 6) samples were considered as mild IDD, and grade IV (*n* = 5) and V (*n* = 5) were considered as the severe IDD group.

### Isolation and culture of human NP cells

Ten mild degenerative disc specimens (Grade II and Grade III) were collected from the patients with IDD and then the NP tissue was carefully separated from the AF under a stereotaxic microscope. The tissue was cut into small fragments, and NP cells were isolated by incubation with 0.25 mg/ml type II collagenase (Invitrogen, Carlsbad, CA, USA) for 12 h at 37°C in Dulbecco's Modified Eagle's Medium (DMEM)/F12 (Gibco, Grand Island, NY, USA). Isolated cells were resuspended in DMEM/F12 containing 10% foetal bovine serum (FBS; Gibco), 100 μg/ml streptomycin, 100 U/ml penicillin, and 1% l-glutamine and incubated at 37°C in a humidified 5% CO_2_ atmosphere. Confluent cells were detached by trypsinization, seeded in 35-mm tissue culture dishes in complete culture medium (DMEM/F12 supplemented with 10% FBS, 100 μg/ml streptomycin, and 100 U/ml penicillin), and incubated at 37°C and 5% CO_2_. The medium was replaced every 3 days. During passaging, no significant changes in morphology of cells between primary cells (passage 0) and later passage cells (passage 2) were noticed. So we used second-passage cells cultured in a monolayer for experiments.

### RNA extraction and quantitative real-time (qRT)-PCR

Total RNA was isolated from NP tissue and cells with TRIzol reagent (Invitrogen) according to the manufacturer's instructions. SOX9, type II collagen, aggrecan, MMP3, MMP13, A disintegrin and metalloproteinase with thrombospondin motif (ADAMTS)4, ADAMTS5, B cell lymphoma (Bcl)-2, and miR-494 expression was quantified by qRT-PCR on a 7500 Realtime PCR System (Applied Biosystems, Foster City, CA, USA) using the cycling conditions recommended by the manufacturer. Primers used for qRT-PCR are listed in Table 1. Reactions were prepared in triplicate. Target gene expression levels were normalized to that of β-actin, and miR-494 level was normalized to that of U6. Relative expression levels were calculated with the 2^−ΔΔCt^ method.

**Table 1:** Sequences of primers used for quantitative real-time PCR

Gene	Oligonucleotide sequence		Product size (bp)
	Forward (5′–3′)	Reverse (5′–3′)	
SOX9	ATGAAGATGACCGACGAGCA	CAGTCGTAGCCTTTGAGCAC	236
Type II collagen	AGAACTGGTGGAGCAGCAAGA	AGCAGGCGTAGGAAGGTCAT	142
Aggrecan	TGAGCGGCAGCACTTTGAC	TGAGTACAGGAGGCTTGAGG	287
MMP3	TTCCTTGGATTGGAGGTGAC	AGCCTGGAGAATGTGAGTGG	248
MMP13	CCCAACCCTAAACATCCAA	AAACAGCTCCGCATCAACC	147
ADAMTS4	ACCCAAGCATCCGCAATC	TGCCCACATCAGCCATAC	246
ADAMTS5	GACAGTTCAAAGCCAAAGACC	TTTCCTTCGTGGCAGAGT	204
Bcl-2	GCCTTCTTTGAGTTCGGTGG	GAAATCAAACAGAGGCCGCA	192
U6	CGCTTCGGCAGCACATATAC	AAATATGGAACGCTTCACGA	100
β-actin	AGCGAGCATCCCCCAAAGTT	GGGCACGAAGGCTCATCATT	285

### Cell transfection

MiR-494 mimic, miR-494 inhibitor, and their negative control (miR-Scr) were designed and synthesized by RiboBio (Guangzhou, China) and transfected into NP cells grown to 80% confluence using Lipofectamine 2000 (Invitrogen) according to the manufacturer's instructions. For *SOX9* knockdown, short interfering (si)RNA against SOX9 (siSOX9) and scrambled siRNA (siScr) (RiboBio) were co-transfected with miR-494 inhibitor or miR-Scr into NP cells using Lipofectamine 2000. After 48 h, cells were harvested and used for analyses.

### Flow cytometry

Apoptotic NP cells were subjected to Annexin V-allophycocyanin (APC)/*7*-amino actinomycin D (7-AAD) double staining and detected by flow cytometry (BD Biosciences, San Diego, CA, USA). Briefly, degenerative human NP cells were harvested after treatment and resuspended in binding buffer. Annexin V-APC and 7-AAD were added to the cells, followed by incubation for 15 min at room temperature.

### MiR-494 target prediction and luciferase reporter assay

TargetScan (www.targetscan.org), miRanda (www.microrna.org), and RNAhybrid (http://bibiserv.techfak.uni-bielefeld.de/) were used to predict miR-494 target genes. SOX9 was found to harbour a putative miR-494 binding site. The luciferase reporter assay was used to determine whether miR-494 directly binds to the 3′-UTR of *SOX9*. The wild-type (WT) or mutant (MUT) 3′-UTR segment containing the putative miR-494 binding site was amplified and inserted into the pGL3 control vector (RiboBio). HEK 293 cells were co-transfected with 100 ng of pGL3 vector harbouring WT or MUT 3′-UTR and 40 nM of miR-494 mimic or miR-Scr using Lipofectamine 2000. After 48 h, cells were harvested and luciferase activity was detected using the Dual Luciferase Reporter Assay System (Promega, Madison, WI, USA). The experiment was repeated three times.

### Western blotting

NP cells were harvested and lysed in radioimmunoprecipitation assay buffer. Protein concentrations were determined with the bicinchoninic acid assay (Beyotime, Shanghai, China). Proteins were separated by 12% sodium dodecyl sulphate polyacrylamide gel electrophoresis and transferred to a polyvinylidene difluoride membrane, which was blocked with 5% non-fat milk and then incubated overnight at 4°C with antibodies against the following proteins: SOX9 (1:1000), type II collagen (1:1000), aggrecan (1:1000), MMP3 (1:1000), MMP13 (1:4000), ADAMTS4 (1:1000), ADAMTS5 (1:500), Bcl-2 (1:1000), and β-actin (1:2000) (all from Abcam, Cambridge, MA, USA); and phospho-NF-κB p65 (Ser536) (1:2000) and NF-κB p65 (1:1000) (Cell Signaling Technology, Danvers, MA, USA). After washing, the membrane was incubated for 2 h at 37°C with horseradish peroxidase (HRP)-conjugated secondary antibody (1:2000; Abcam). Protein bands were visualized by enhanced chemiluminescence (Pierce, Rockford, IL, USA). β-actin was used as a loading control. The experiment was repeated three times.

### Immunofluorescence staining

Cultured NP cells were washed three times with phosphate-buffered saline (PBS), fixed with 4% formal dehyde for 15 min at 37°C, and blocked with 3% bovine serum albumin (BSA) for 30 min. The cells were then incubated overnight at 4°C with a primary antibody against type II collagen (1:150), followed by Cy3-conjugated goat anti-rabbit IgG antibody (1:100; Abcam) for 2 h at room temperature. Nuclei were counterstained with diamidino-2-phenylindole (Beyotime), and cells were visualized using a fluorescence microscope (Olympus, Tokyo, Japan).

### Immunohistochemistry

Human NP tissue samples were fixed with 4% paraformaldehyde for 24 h, embedded in paraffin, and cut into sections that were deparaffinized, rehydrated, and treated with trypsin for 60 min at 37°C. Endogenous peroxidase activity was quenched by treatment with 3% H_2_O_2_ for 15 min at room temperature. The sections were blocked with 3% BSA for 30 min at room temperature, then incubated overnight at 4°C with primary antibody against SOX9 (1:100). After incubation with HRP-conjugated secondary antibody (1:3000), immunoreactivity was detected by treatment with diaminobenzidine in PBS. Sections were counterstained with haematoxylin and imaged with a microscope (Olympus).

### Enzyme-linked immunosorbent assay (ELISA)

The level of MMP-13 secreted by NP cells in the culture supernatant were measured using MMP13 ELISA kit (R&D Systems, Inc., Minneapolis, MN, USA) according to the manufacturer's instructions.

### DMMB assay

To quantify the sulfated glycosaminoglycan (sGAG) content synthesized by NP cells, the 1,9-dimethylmethylene blue (DMMB) assay was performed as previously described [48] using a Blyscan sGAG Assay kit (Biocolor Ltd., Carrickfergus, UK) according to the manufacturer's instructions.

### Methylation-specific PCR (MSP) and bisulfite sequencing PCR (BSP)

The methylation status of the miR-494 promoter in NP samples was examined by MSP and BSP. Genomic DNA was extracted using a DNA Extraction kit (Promega). Sodium bisulfite conversion was carried out using the Epitect Bisulfite kit (Qiagen, Hilden, Germany). MSP was carried using primers recognizing the methylated and unmethylated forms of the promoter. The forward and reverse primer sequences were as follows: methylated, 5′-TAAAAGTAATTTGGGATACGTAAAT-3′ and 5′-AAA AAAAACAACACGAACAAC-3′; and unmethylated, 5′-TAAAAGTAATTTGGGATATGTAAAT-3′ and 5′-AA AAAAACAACACAAACAAC-3′. MSP products were separated on a 3% agarose gel containing ethidium bromide and visualized under ultraviolet illumination. Forward and reverse BSP primers were designed using Methprimer (http://www.urogene.org/methprimer/) and had the following sequences: 5′-GATTTTTTAA AAGTAATTTGGGATA-3′ and 5′-CATCATAAATAAAAAAAAAACAACAC-3′. BSP products were subcloned into pMD19-T vector (Takara Bio, Otsu, Japan) according to the manufacturer's instructions. Ten clones of each sample were sequenced by Invitrogen (Shanghai, China).

### Treatment with 5-aza-2′-deoxycytidine (5-aza-CdR)

Mild IDD NP cells were treated with 5 μM of the demethylation agent 5-aza-CdR (Sigma-Aldrich, St. Louis, MO, USA) for 3 days. Medium containing the agent was replaced daily, and cells were harvested for analysis.

### Statistical analysis

Data are expressed as mean ± standard deviation of at least three independent experiments. Statistical analyses were performed using SPSS v.18.0 software (SPSS Inc., Chicago, IL, USA). Mean differences between groups were evaluated with the Student's *t* test or by analysis of variance. The Pearson correlation coefficient was used to assess the correlation between miR-494 and *SOX9* expression. *P* values < 0.05 were considered statistically significant.
